# Rodent Models of Amyloid-Beta Feature of Alzheimer’s Disease: Development and Potential Treatment Implications

**DOI:** 10.14336/AD.2019.1026

**Published:** 2020-10-01

**Authors:** Chi Him Poon, Yingyi Wang, Man-Lung Fung, Chengfei Zhang, Lee Wei Lim

**Affiliations:** ^1^School of Biomedical Sciences, Li Ka Shing Faculty of Medicine, The University of Hong Kong, Hong Kong, China.; ^2^Endodontology, Faculty of Dentistry, The University of Hong Kong, Hong Kong, China

**Keywords:** Alzheimer’s disease, amyloid-beta, neuroinflammation, neuroplasticity, neurotrophic factors

## Abstract

Alzheimer’s disease (AD) is the most common neurodegenerative disorder worldwide and causes severe financial and social burdens. Despite much research on the pathogenesis of AD, the neuropathological mechanisms remain obscure and current treatments have proven ineffective. In the past decades, transgenic rodent models have been used to try to unravel this disease, which is crucial for early diagnosis and the assessment of disease-modifying compounds. In this review, we focus on transgenic rodent models used to study amyloid-beta pathology in AD. We also discuss their possible use as promising tools for AD research. There is still no effective treatment for AD and the development of potent therapeutics are urgently needed. Many molecular pathways are susceptible to AD, ranging from neuroinflammation, immune response, and neuroplasticity to neurotrophic factors. Studying these pathways may shed light on AD pathophysiology as well as provide potential targets for the development of more effective treatments. This review discusses the advantages and limitations of these models and their potential therapeutic implications for AD.

## Introduction

Alzheimer’s disease (AD) has a high incidence rate affecting nearly 40 million people worldwide, which is predicted to increase in the coming years [1]. It is one of the most common causes of senile dementia in Europe and America, and is estimated to account for 50%-80% of all senile dementia cases worldwide [2]. In 2015, AD resulted in 110,561 deaths in Americans above 65 years of age, which was the sixth leading cause of death in the United States. In 2017, more than 16 million family caregivers provided an estimated 18.4 billion hours of care to patients with Alzheimer’s and other dementias at an estimated cost of over $232 billion, not to mention the intangible cost of the physical and psychological stress experienced by these carers [3]. Symptoms of AD include progressive cognitive decline, which is characterized by initial memory loss that gradually progresses to cognitive impairments and behavioral changes. Generally, AD patients do not present with clinical symptoms until the dementia stage, which makes early diagnosis difficult and makes the clinical study of AD pathogenesis challenging.

During the past 20 years, the identification of genes and proteins related to AD has led to the development of transgenic rodent models. These rodent models play an important role in the elucidation of the underlying disease mechanisms of AD, and significant progress has been made in understanding the neurobiological basis of AD. These transgenic rodent models are also useful for testing the effects of potential therapeutic novel drugs and interventions. However, the heterogeneous nature of AD hinders the development of effective therapeutics. It is well known that the main neuropathological hallmarks of AD include amyloid plaques and neurofibrillary tangles, as well as the loss of certain neurons, especially cholinergic, noradrenergic, serotonergic, and pyramidal neurons [4]. Amyloid plaques are composed of dystrophic neurites surrounded by a central core, whereas neurofibrillary tangles are composed of highly phosphorylated microtubule-associated tau proteins in specific neuronal perikarya. In the past decades, a deeper understanding of neurobiological basis of the etiology and pathogenesis AD involving neuroprotection, neuroplasticity, and anti-neuroinflammation pathways has led to the rapid development of potential treatments based on these pathways.

Neuron loss can occur in the early stage of AD even before senile plaques and neurofibrillary tangles have developed [5]. Loss of neurons is regarded as one of the causes of brain function decline. Compared to normal aging, neuron death in AD was observed to be more intense in certain brain areas [6, 7]. In the early stage of AD, the neuron loss starts in hippocampal area CA1, dentate fascia, subiculum, and layer 2 of entorhinal cortex [5, 8-10], which then spreads into the temporal, frontal, and parietal lobes of the cerebral cortex [6]. In the late stage of AD, neuron loss is observed in the whole brain including olfactory bulbs [11], amygdala, basal nucleus of Meynert [12, 13], substantia nigra [14], locus coeruleus [15], and dorsal raphe nucleus [16]. Decreased neuron numbers in the CA1 and entorhinal cortex was associated with memory impairment [17]. The decline in the number of neurons is caused by the disruption of normal physiological processes including postnatal neurogenesis and natural neuronal death. These alterations could also result from suppressed maturation and functional integration of new born neurons in the dentate gyrus [18], as well as reduced postnatal neurogenesis occurring in the subventricular area and dentate gyrus [19, 20]. However, the exact molecular mechanism that induces neuron loss in AD has not yet been established.

Neurofibrillary tangles are considered to be one of the major pathological features of AD and the main microscopic lesion. These abnormal fibrous inclusions were first discovered in 1907 by Alois Alzheimer in perikaryal cytoplasm of pyramidal neurons [21]. The neurofibrillary tangle has been widely studied in the past decades. On a ultrastructural level, the neurofibrillary tangle consists of abnormal fibrils approximately 10 nm in diameter, which appear as helically wound pairs of filaments with a periodicity of 80 nm [22, 23]. The main component of the neurofibrillary tangle is hyper-phosphorylated microtubule-associated Tau protein, which is located on specific molecular sites in the neurofibrillary tangles [24]. Although the neurofibrillary tangles contain many other proteins including cholinesterases [25], ubiquitin [26, 27], and beta-amyloid 4 [28], Tau is regarded as the most important component. In clinical cases of AD, neurofibrillary tangles were generally found in most brain areas and its distribution mode is relatively predictable. Severe neurofibrillary tangles were observed in the layer 2 neurons of the entorhinal cortex, CA1 and subiculum regions of the hippocampus, amygdala, and deeper layers (layers III, V, and superficial VI) of the neocortex in AD brain [29]. The range and location of neurofibrillary tangles in AD patients are associated with the degree of dementia and the stage of disease [30, 31], indicating these pathological features may be involved in the disruption of brain function.

Senile or neuritic plaques are also considered to be a main pathological feature of AD. Senile plaques are a common pathologic feature found in post-mortem AD patients [32] and AD animal models [33]. Several forms of amyloid-beta (Aβ) plaques are also observed in aged people as well as in AD patients. The main component of these plaques is beta-amyloid A4 (βA4) protein located in the central core, which displays a radial arrangement and is surrounded by abnormally generated neurites or neuronal processes [34]. It is more common to find neuritic plaques surrounded by several microglial cells, instead of reactive astrocytes [34]. Currently, there are ongoing debates regarding the involvement of microglia in the neuroinflammatory pathogenetic cascade and in the reaction to components within lesions [35]. The βA4 protein is generated from a larger transmembrane glycoprotein called amyloid precursor protein (APP) [36]. Two secretases split the amino and carboxyl terminals of the 4-kD segment to generate the βA4 fragment [37-39]. Interestingly, due to irregular cleavage of the carboxyl end, this process can generate products with different lengths. Depending on the lengths, these products can either accumulate within senile plaque (42 or 43 amino acids) or deposit within the leptomeningeal, cerebral cortical, and cerebellar blood vessels (40 amino acids) [40].

### Amyloid-beta pathology in mouse models

Based on the current evidence, Aβ is a significant pathological factor with many established associations with other pathologies present in AD. In 1992, Hardy and Higgins [41] first proposed the amyloid cascade hypothesis, which has been gradually modified in the past two decades. The amyloid cascade hypothesis is considered to be one of the dominant theories in AD and has gained much support [42]. This hypothesis is important for AD research as it postulates that Aβ facilitates the progression of AD and leads to other downstream phenotypic manifestations such as cognitive declines and Tau pathology. Furthermore, *in-vitro* and transgenic animal model experiments showed that intraneuronal accumulation of Aβ precedes the pathological manifestations of AD and contributes to various pathological effects on cellular functions [43].

Given the importance of Aβ in AD pathogenesis, this review focuses on the use of transgenic rodent models to study beta-amyloid in AD pathogenesis. Transgenic rodent models of Aβ accumulation or amyloid plaque formation can mimic many aspects of human AD and have provided much evidence to support the amyloid cascade hypothesis. These rodent models are, therefore, invaluable as tools for exploring the pathological processes in AD and the relationship between AD pathology and the cerebral microenvironment.

With the identification of genes associated with AD progression such as amyloid precursor protein, presenilin 1 (PS1), and presenilin 2 (PS2), several mouse models based on these mutations have since been generated [44]. These transgenic rodent models have been important tools for clarifying the underlying mechanisms of AD and to explore biomarkers of the early stage AD. Unlike humans, rodents do not develop Aβ plaques in the natural aging process, which could be due to differences in the three Aβ sequences across species [45]. Several mouse models have been generated with genetic mutations related to human amyloid precursor protein (APP), C-terminal fragment of APP [46], Aβ, and familial forms of Alzheimer’s disease [47-50], which result in robust Aβ accumulation.

**Figure 1.**
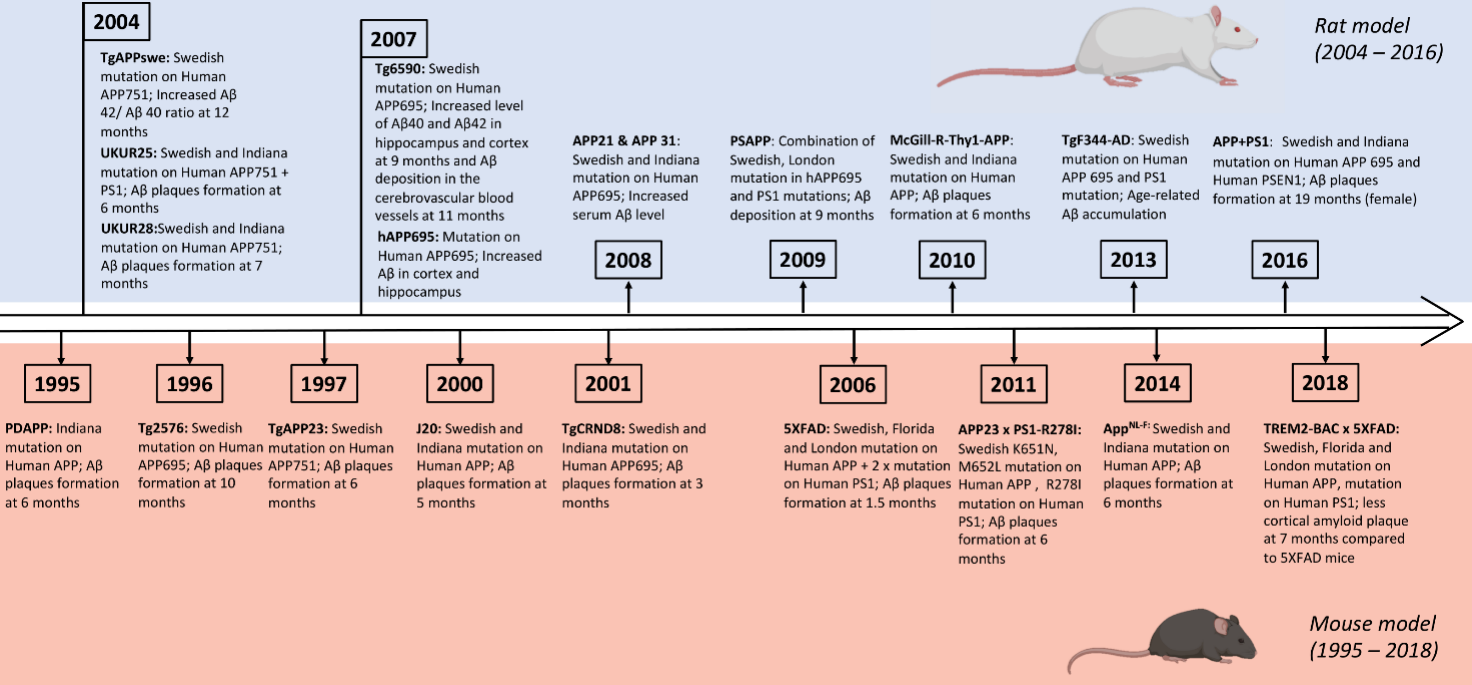
Timeline illustrating the development of transgenic mouse and rat models of AD.

### Transgenic mouse models with human APP mutation

The most common approach to generate transgenic AD mouse models is to overexpress the Familial Alzheimer’s disease (FAD)-related human APP mutation. This transgenic AD mouse model exhibits rapid disease manifestation and plaque formation and has been widely used in many research studies. Nowadays, more than 50 transgenic mouse models are used in this field of research, and the majority of them are characterized by the overexpression of human wildtype or FAD-related mutant APP.

The first mouse models were generated by overexpressing human wildtype APP. However, these mice had only mild neuropathological manifestations and did not exhibit Aβ plaques, indicating a failure to effectively mimic human AD [51-53]. Subsequently, mouse models generated with FAD-related APP mutations gradually developed the molecular pathology. Mice with these APP mutations manifested an age-related increase and maturation of amyloid plaques in the brain. The minimum age of the formation of amyloid plaques in mutant mice was found to be closely associated with the chosen mutations and transgenic expression promoters that govern the transcript level of transgenes in the brain. Notably, the majority of transgenic mice models expressing mutant human APP showed an age-dependent cognitive decline resembling that of human AD [54-56]. These mutant mice also displayed some neuropathological features of human AD, such as dystrophic neurites, reactive astrocytes, activated microglia, increased innate immunity and inflammation, synapse loss, and disrupted electrophysiological and neurochemical signaling [57]. However, only a few of these transgenic mice exhibited significant neural degeneration or neurofibrillary tangle (NFT)-like tau [57], suggesting overexpression of FAD-related APP mutations is not sufficient to completely mimic human AD.

A common mutation in transgenic AD mice is Swedish double APP mutation, with about half of the APP transgenic mice models overexpressing this mutation. A widely used model is the Tg2576 mouse model, which possesses the Swedish mutation driven by the hamster prion promoter that leads to transgene expression in the forebrain area and spinal cord [58]. These mice have thioflavin-S-positive Aβ plaques at the age of 10-12 months, and also generate oligomeric Aβ, a toxic component of the plaque [59]. Although Tg2576 mice did not show significant neuronal loss in brain areas [60], they did have impaired dendritic spine stability, leading to severe spine loss and reduced synaptic plasticity [61]. Nevertheless, it is not known whether synaptic loss or cognitive declines are consequences of the formation of Aβ oligomers, fibrils, or plaques.

Another APP transgenic mouse model, TgAPP23, was generated using a longer 751 amino acid version of APP with the expression driven by promoter murine Thy-1.2. This mouse model exhibited amyloid deposition at the age of 6 months, with the severity escalating by 12 months particularly in brain vessels, leading to gradually reduced blood flow and altered vessel morphology [62, 63]. These mice also showed reactive gliosis, astrocytosis, dystrophic neurites, synaptic loss, and even neuron loss in the hippocampal area CA1 at 14-18 months [64].

The PDAPP mouse model expressing a human Indiana mutation of APP minigene was driven by the promoter of platelet-derived growth factor [54, 65]. Compared to other mouse models, PDAPP mice had age-dependent diffuse and dense Aβ plaques at the age of 6-9 months. These mice also showed reactive astrocytes around these Aβ plaques and significant synaptic loss with age, but no evidence of neuronal loss in AD pathology-related brain areas such as entorhinal cortex and hippocampal area CA1 [54, 66].

Taken together, these findings suggest the progress of Aβ pathology in these mice is largely dependent on the expression of the transgene and formation of specific Aβ species in the brain. Thus, another strategy to facilitate Aβ pathology is to combine several FAD-related mutations. Generally, the combination of Swedish double mutations and APP transgene mutations accelerated the generation of toxic Aβ42 peptides, resulting in a more rapid and robust presentation of Aβ pathology compared to the above transgenic mouse models. For instance, J20, APP22, and TgCRND8 mouse models with several FAD-related mutations exhibited more robust Aβ pathology [53, 56, 67]. The J20 mice displayed diffuse amyloid deposits in the hippocampus and neocortex at the age of 5-6 months and subsequently showed large neuritic plaques by 9 months [68]. These mice also showed a decline in synaptophysin immunoreactivity, suggesting altered synaptic function [69]. The J20 mice exhibited significant deficits in spatial learning and memory, suggesting these molecular pathologies may translate to cognitive impairments [70]. Another mouse model, TgCRND8, with multiple FAD-related mutations showed even earlier onset of pathologies, with significant cognitive impairment and Aβ plaque deposition at 3 months [67], and activation of immune or inflammatory reaction and cholinergic input loss at 7 months [71]. These findings indicate that multiple transgenes can contribute to an accelerated Aβ pathology.

The current evidence derived from APP transgenic mice and clinical research indicates that Aβ oligomers, instead of plaques, may lead to the clinical phenotypes observed [72]. A new APP transgenic mouse, APP_E693_Δ-Tg, provided further evidence that Aβ oligomers play an important role in AD pathology and cognitive deficits. This mouse model did not display any extracellular Aβ plaques, but had increased accumulation of soluble Aβ oligomers in neurons [73]. At the age of 8 months, they exhibited synaptic loss and increased phospho-tau in mossy fibers, which are associated with the accumulation of Aβ oligomers [57]. At an older age of 18-24 months, they exhibited gliosis and neuronal loss in hippocampal area CA3 [57]. These results support the notion that Aβ oligomers, instead of plaques, may be a crucial component that triggers neurotoxicity and cognitive impairment in AD. This phenomenon resembles the observations from clinical studies in AD patients that showed a link between cerebral Aβ levels and cognition [74, 75]. However, further research is needed to elucidate the specific Aβ oligomer species that may be more pathogenic in this disease.

To interpret the phenotypes in APP transgenic mouse models, it is necessary to untangle the effects of Aβ over those from APP overexpression. To avoid potential artifacts due to APP overexpression, the *App*^NL-F^ mouse model was created to augment Aβ42 without altering the APP expression level [76]. Specifically, this APP knock-in mouse includes a humanized Aβ region with two Swedish and Iberian mutations [76]. This model exhibited Aβ accumulation starting from an early age, likely resulting from increased Aβ42. The integrated effects of APP proteolysis of Swedish and Iberian mutations led to elevated total Aβ and Aβ42, respectively [76]. The initial formation of amyloid plaques in the *App*^NL-F^ mouse model was detected at 6 months [76]. Impairment of the working memory was observed at 18 months as detected in the Y maze, but there was no significant impairment in spatial learning and memory as detected by the Morris water maze (MWM) [76].

### Transgenic mice with Presenilin mutations

In addition to APP mutations, previous evidence indicates that PS mutations, of which there are more than 200 mutations identified so far, also contribute to FAD pathology. PS mutations have been shown to cause an alteration of γ-cleavage in APP, which forms more amyloidogenic Aβ42 peptides, thereby increasing the ratio of Aβ42:Aβ40 in the brain. Transgenic mice with mutant PS1 or PS2 were generated to study which PS mutations are implicated in AD pathology. Interestingly, although they exhibited AD pathological phenotypes, these models failed to develop amyloid plaques in their brains. The reduced Aβ aggregation in these mouse models may be attributed to differences in Aβ sequences between mice and humans [45]. Some PS1 mutant mouse models showed age-related neurodegeneration in the CA1 region and synaptic loss in the stratum radiatum region of the hippocampus [77-79]. The PS1 L286V transgenic mice, which displayed intracellular accumulation of Aβ40 but lacked plaque development, had increase intracellular accumulation of Aβ42 at 17-24 months [77].

Double transgenic mouse models have been widely used in the study of Aβ pathology and evaluation of anti-amyloid treatments. Double transgenic mouse models that exhibit sufficient Aβ42 and have Aβ plaque formation at an early age were generated by crossing human APP and presenilin transgenic lines [80]. Another important example of APP/PS1 double transgenic mouse model is 5XFAD mice, which co-express five FAD mutations. The 5XFAD mouse model can almost exclusively produce Aβ42 and shows rapid accumulation of high levels of Aβ42 in the brain [81]. These mice had intraneuronal Aβ42 accumulation at the age of 1.5 months and subsequent amyloid deposition and gliosis, particularly in the subiculum and deep cortical layers, at 2 months [81]. Moreover, they also had significant neurodegeneration and neuronal loss, which appeared to be associated with the intraneuronal Aβ and amyloid plaques, respectively [81]. To investigate amyloidogenic Aβ43, another double transgenic mouse model was created by crossing APP transgenic mouse with PSA-M146V knock-in mouse. Interestingly, this mouse model showed an early increase in Aβ43 levels at 3 months, as well as augmented Aβ42 and Aβ40 levels at 9 months. The APP23 x PS1-R278I mouse model exhibited robust progressive plaque deposition starting from the age of 6 months [82]. The APP23 x PS1-R278I mouse model also displayed short-term memory decline at 3-4 months as shown by the Y maze, although spatial learning and memory remained largely intact [82]. These transgenes introduction contributed significantly to Aβ pathology, as shown in the increased ratio of Aβ peptides and elevated Aβ43, followed by accelerated plaque deposition and cognitive impairment, suggesting alterations of these Aβ peptides may be deleterious in AD.

To investigate the effects of some specific genes, the 5XFAD mouse model was crossed with other mouse lines to establish new mouse models. For example, the TREM2-BAC x 5XFAD mouse model was generated to probe TREM2, a microglia-enriched gene associated with a recessive disease named Nasu-Hakola disease, which is characterized by bone cysts and early dementia [83]. In addition, a subset of loss-of-function TREM2 variants was found to predispose a frontotemporal dementia-like syndrome in the absence of apparent bone involvement [84]. Given the shared disease manifestations, TREM2 biology in microglia may be involved in AD pathogenesis. To study the effect of the TREM2 gene in AD pathogenesis, several mouse models were generated including TREM2-BAC x 5XFAD [85], TREM2 Humanized (common variant) x 5XFAD [86], and TREM2 Humanized (R47H) x 5XFAD [86]. The TREM2-BAC x 5XFAD mice produced less cortical amyloid plaques than 5XFAD mice at 7 months and had similar performance in contextual fear conditioning tests compared to wildtype mice, indicating that TREM2 expression may somehow reduce the Aβ pathology and alleviate cognitive decline in AD [85].

Other than double transgenic mouse models, which encompass mutations in two of the genes of interest in AD: APP and PS1 genes, a 3xTg-AD mouse model was generated to include mutations of human Tau (P301L), APP695 (KM670/671NL) and PS1 (M146V) [87]. The 3xTg-AD mice exhibited severely impaired long-term potentiation, which was thought to be mediated by intracellular Aβ accumulation at the age of 6 months [87]. Such Aβ pathology was wide spread across brain regions (hippocampus, amygdala, frontal cortex, thalamus), and was also demonstrated to underlie the behavioral deficits observed in contextual fear conditioning and MWM, as Aβ clearance enhanced by the administration of anti-Aβ antibody successfully reversed the long-term spatial memory impairment observed in MWM [88]. A recent study further confirmed 6-month old as the age of onset for cognitive deficits and signs of neuroinflammation as represented by a significantly higher degree of microglia activation [89]. Although 3xTg-AD mice carry mutations in three different genes that were thought to contribute synergistically to the neuropathology of AD [90, 91], the fact that Aβ deposits were observed prior to neurofibrillary alterations suggests that Aβ pathology remains the major driving force of disease manifestation in AD.[Table T1-ad-11-5-1235]

**Table 1 T1-ad-11-5-1235:** Mouse models of amyloid-beta pathology in Alzheimer’s disease.

Animal model	Model background	Transgene	Amyloid pathology	Other pathology	Behavioral test	Ref.
Tg2576	C57BL/6	Human APP695 (Swedish)	Aβ plaques at 10-12 months,oligomeric Aβ generation	Synaptic loss at 15-18 months.	Behavioral impairment in novel object recognition at 12-15 months, Morris water maze at 6 months and Y maze at 10 months.	[55, 123, 237]
TgAPP23	C57BL/6J	Human APP751 (Swedish)	Aβ plaques at 6 months	Increased level of phospho-tau at 6 months, phospho-tau deposition surrounding plaques at 12 months, neuronal loss in area of CA1 at 14-18 months.	Behavioral impairment in novel object recognition at 3-4 months, Morris water maze at 3 months.	[56, 238, 239]
PDAPP	Swiss Webster × B6D2F1	Human APP (Indiana)	Aβ plaques at 6-9 months	Synaptic loss.	Behavioral impairment in novel object recognition at 6 months, Morris water maze at 3 months.	[54, 240]
J20	C57BL/6 × DBA/2 F2	Human APP (Swedish and Indiana)	Diffuse amyloid deposits at 5-6 months and larger neuritic plaques at 9 months	Phospho-neurofilaments.	Behavioral impairment in novel object recognition at 4 months, Morris water maze at 6-9 months.	[53, 68, 241, 242]
TgCRND8	C3H/He × C57BL/6	Human APP695 (Swedish and Indiana)	Aβ plaques at 3 months,dense core plaques at 5 months, spreading to the cerebellum and brainstem by 8-9 months	Astrocytic gliosis and microglial activation in regions around plaques.	Behavioral impairment in novel object recognition at 3-5 months, Morris water maze at 3 months.	[67, 69, 243]
*App*^NL-F^	C57BL/6	Human APP (Swedish and Iberian)	Aβ plaque at 6 months	Synaptic loss, microgliosis and astrocytosis	Behavioral impairment in Y-maze at 18 months, no impairment in Morris water maze at 18 months	[76]
5XFAD	Tg6799×Tg7031 ×Tg7092	Human APP (Swedish, Florida, London); Human PS1 (M146L, L286V)	Intraneuronal Aβ42 accumulation at 1.5 months, amyloid deposition, gliosis, at 2 months	Significant neurodegeneration and neuronal loss.	Behavioral impairment in Y-maze at 4-5 months, decreased Interest in social-related behaviors at 3-12 months. Morris water maze at 4 months.	[81, 244, 245]
APP23 x PS1-R278I	C57BL/6J	Human APP23 (Swedish K651N, M652L); Human PS1 (R278I)	Aβ plaque at 6 months	Astrocytosis.	Behavioral impairment in Y-maze at 3-4 months; no significant impairment in Morris water maze	[82]
TREM2-BAC x 5XFAD	TREM2-BAC: FVB/NJ; 5XFAD: C57BL/6X SJL	Human APP (Swedish, Florida, London); Human PS1 (M146L, L286V)	less cortical amyloid plaque at 7 months compared to 5XFAD mice	Enhanced process ramification and phagocytic marker expression in plaque-associated microglia; improved dystrophic neurites.	No cognitive impairment in contextual fear conditioning test.	[85]
3xTg-AD	C57BL6/129SvJ	Human APP (Swedish); Human PS1 (M146V); Human Tau (P301L)	Aβ plaque at 6 months	synaptic dysfunction and increased microglia activation at 6 months; Tau alteration at 12-15 months	Retention deficits in Morris water maze and contextual fear memory	[87-89]

## Amyloid-beta pathology in rat models

### UKUR25 and UKUR28 rats

The first transgenic rat model of AD displayed intracellular amyloid-beta accumulation but without senile plaques. The UKUR25 model with human APP 751 and PS1 transgenes and the UKUR28 model with mutated human APP 751 transgene were both reported to display intracellular accumulation of Aβ peptides in the cerebral cortex and hippocampus at the age of 6 months corresponding to the “pre-plaque” stage in AD [92]. Interestingly, aged rats in both transgenic lines did not have senile plaques at 24 months [92]. One possible explanation of such findings was that the Aβ levels were not sufficient to initiate the deposition and subsequent senile plaque formation. As shown by behavioral tests, both transgenic models displayed a mild decline in spatial acquisition learning at 16 months [93]. Downstream molecular analysis revealed that intracellular accumulation of Aβ could induce the activation of the mitogen-activated protein kinase (MAPK) ERK2 and phosphorylation of tau proteins at the PHF-tau epitope [92], indicating that Aβ peptides may be an essential component that drives the diverse pathogenesis of AD.

### APPswe rat

The first APP transgenic rat model, APPswe, was created in 2004 by Ruiz-Opazo [94]. To generate the APPswe rat model, Swedish mutated APP expression driven by the platelet-derived growth factor promoter was introduced into a background strain of Fisher-344 rats [95]. The transgenic APPswe rats displayed increased intracerebral APP mRNA and elevated levels of Aβ42 and Aβ40 peptide at 12 months [94]. Surprisingly, 6- and 12-month-old APPswe rats demonstrated enhanced cognitive performance in hippocampus-dependent behavioral tests, including MWM and Social Transmission of Food Preference (STFP) task [94]. These results indicate that APP and its derivatives may have potential roles in learning and memory functions. Further research on this transgenic model may provide insights on the effects of intracerebral APP level on memory.

### Tg6590 rat

The transgenic Tg6590 rat expressing the human APP with Swedish mutation was developed in 2007 by Folkesson [96]. Molecular characterization of this rat model showed increased levels of Aβ40 and Aβ42 in the hippocampus and cortex at 9 months, and subsequent Aβ deposition mainly in the cerebrovascular blood vessels at 11 months [97]. Notably, 9-month-old Tg6590 rats showed impaired spatial learning and memory in the MWM and reduced exploratory behaviors in the Open Field Test (OFT), which manifested prior to Aβ deposition [97]. The observation of behavioral deficits preceding the onset of Aβ pathology indicates that Aβ formation may not be necessarily linked with cognition. More investigations are needed on the transgenic Tg6590 rat model to fully understand the importance of this finding in AD pathology.

### hAPP695 rat

*The hAPP* isoform 695(*hAPP*695)is recognized as a specific APP isoform preferentially expressed by neurons. Previous clinical studies showed reduced APP695 transcripts in post-mortem brains of AD patients [98]. To further elucidate the role of this isoform in AD pathology, its gene was introduced into the genome of wildtype rats to generate the *hAPP*695 model. The transgenic *hAPP*695 rat had a two-fold increase in APP level in the hippocampus and cortex [99], indicating this transgene partly mediates Aβ pathology in AD. Further behavioral characterization of *hAPP695* rats showed impaired locomotor and spatial learning and memory after middle cerebral artery occlusion [99]. Moreover, a few studies suggested that enhanced APP/Aβ may lead to altered brain function, such as impaired skill [100] and spatial learning [101], and was possibly associated with negative effects on cognition. This further substantiated the detrimental effects of transgene mutation / introduction in AD animal models. Interestingly, previous evidence showed that APP and/or Aβ accumulation was increased in rodents after inducing ischemia [102, 103], which may imply a possible compensation effect of APP/Aβ from brain damage. Therefore, the aforementioned findings from *hAPP*695 rat may offer a different opinion on the effect of APP/Aβ on brain structural damage [99].

### APP21 and APP31 rats

Two APP-transgenic rat lines, APP21 and APP31, were generated using Fischer 344 rats by lentiviral vector infection of Fischer 344 zygotes. In a previous study, APP21 rats were found to have a serum Aβ42 level of 135 pg/mL, and both APP21 and APP31 rats had APP mRNA levels that were 7.6 and 3 times more than in wildtype rats, respectively [104]. Additionally, the immune-histochemistry showed the human APP transgene was expressed in neurons, but not in glial cells [104]. The transgenic APP21 rats showed impaired spatial memory in the MWM test at an early age of 3 months, but did not show deteriorated long-term memory recall ability with age, similar to that of wildtype rats [105].

### PSAPP rat

In 2007, Flood et al. reported a series of new transgenic rat models that were generated by crossing two Sprague-Dawley rat lines with different transgene expressions of human APP [106]. This transgenic process gave rise to rat models that expressed single, double, or triple transgenes denoted by their carried mutations: Tg478 rats expressed human APP with Swedish mutation driven by rat synapsin promoter, Tg1116 rats expressed a human APP minigene with Swedish and Indiana FAD mutations, and Tg11587 rats expressed Swedish and Finnish FAD mutations. The resulting double homozygous rats displayed adequate levels of Aβ accumulation to form amyloid deposition at the age of 17 to 18 months. Crossing with a third transgenic rat carrying a human PS-1 transgene with the familial AD mutation M146V (Tg11587) reduced the progression time, with Aβ deposition observed at 7 months. A triple homozygous transgenic rat, which included three transgene expressions (Tg478/Tg1116/Tg11587), also known as PSAPP rat, had Aβ deposition similar to that found in mouse models [107]. The PSAPP rat also displayed cognitive impairment in the MWM at 7 months, but showed no changes in anxiety and locomotion in the elevated plus maze and OFT [107]. However, PSAPP rats overexpressing numerous transgenes are vulnerable to many diseases such as chronic kidney disease and hypertension [108].

### TgF344-AD rat

The transgenic (Tg) F344-AD rat model expresses both human APP (APPswe) and PS1 (PS1ΔE9) genes, which are both independent factors in early-onset familial AD. This rat model was found to have age-related cerebral Aβ accumulation that induced a tauopathy, together with cognitive impairment, apoptosis, and neuron loss [109]. The TgF344-AD rat exhibited reversal learning declines in the MWM at 6 months [110] and learning deficits in the Barnes maze at 15 months [109]. Compared with other models that had limited neuron loss, TgF344-AD rats displayed persistent and severe neuron loss in the cortex and hippocampus, together with shrinking hemispheric brain and spongiform-like vacuolar pathology of aged rats [109]. The NFT pathology in TgF344-AD rats was found to involve endogenous rat tau protein compared to animal models of AD based on the mutated human tau transgene [109]. Overall, these results demonstrated that the TgF344-AD rat model had significant progressive neurodegeneration, further supporting this rat model as a promising animal model for the future AD research.

### McGill-R-Thy1-APP rat

The transgenic McGill-R-Thy1-APP rat expresses human APP with Swedish and Indiana mutations driven by the murine Thy1.2 promoter. The McGill-R-Thy1-APP rat showed a gradual increase in Aβ accumulation in the cortex and hippocampus at 1 week postnatally [111]. Amyloid plaque formation associated with the activation of glial cells and dystrophy of surrounding neurites was detected at 6 months [111]. Interestingly, despite the absence of plaque generation at 3 months, this rat model exhibited spatial learning and memory impairments in the MWM dependent on the soluble cortical Aβ level, which became more severe at 13 months [111]. Another study on the McGill-R-Thy1-APP rat showed similar findings, which supports the validity of this transgenic rat model. These transgenic rats exhibited spatial cognitive deficiency at 4, 6, and 12 months. Moreover, the rats were observed to have working memory decline in the Y maze, as well as higher anxiety levels in the OFT at 6 and 12 months [112].

### APP+PS1 rat

The APP+PS1 rat expressing human APP with Swedish and Indiana mutations and human PS1 with L166P mutation was generated by injecting a lentiviral vector containing PSEN1 into the fertilized egg of homozygous APP21 rats [113]. Compared to APP21 rats, APP+PS1 rats had approximately two times the level of Aβ and more severe amyloid pathology, as well as memory and learning deficits that resembled AD [113]. At 10 months, APP+PS1 rats displayed cognitive declines in various areas such as acquisition, retention, and reversal phase as shown in the Barnes maze task [113]. At 19 months, female APP+PS1 rats were found to have amyloid deposits and cerebral amyloid pathology [114].

## Advantages of rodent models in AD

Given the ethical considerations as well as the late manifestation of symptoms in AD patients, clinical investigation of AD pathogenesis is challenging. Transgenic rodent models of AD could be an effective alternative approach for studying the pathological processes. Furthermore, animal models allow us to perform in-depth terminal studies in different age groups for a better understanding of disease progression and the molecular mechanisms contributing to the pathogenesis. Models that successfully mimic the pathogenesis of AD could also be employed to screen potential therapeutic agents in preclinical settings. Transgenic animal models can also be used in animal behavior testing to validate treatment efficacy. The use of transgenic animal models for disease research also provides translational value. For instance, transgenic mouse models were shown to be phylogenetically conserved with humans in terms of the architecture and function of the hippocampal and entorhinal cortex circuits, sharing similar numbers of genes and considerable chromosomal synteny. Despite the difficulty in generating transgenic rat models, they can be a viable option in the research of highly conserved physiological, morphological, and genetic conditions similar to human diseases [115-117]. For example, the apoE amino sequences between human and rat are quite homologous, sharing 73.5% and 73.9% of human apoE3 and apoE4, respectively [118, 119].[Table T2-ad-11-5-1235]

**Table 2 T2-ad-11-5-1235:** Rat models of amyloid-beta pathology in Alzheimer’s disease.

Animal model	Model background	Transgene	Amyloid pathology	Other pathology	Behavioral test	Ref.
Tg2576	C57BL/6	Human APP695 (Swedish)	Aβ plaques at 10-12 months,oligomeric Aβ generation	Synaptic loss at 15-18 months.	Behavioral impairment in novel object recognition at 12-15 months, Morris water maze at 6 months and Y maze at 10 months.	[55, 123, 237]
TgAPP23	C57BL/6J	Human APP751 (Swedish)	Aβ plaques at 6 months	Increased level of phospho-tau at 6 months, phospho-tau deposition surrounding plaques at 12 months, neuronal loss in area of CA1 at 14-18 months.	Behavioral impairment in novel object recognition at 3-4 months, Morris water maze at 3 months.	[56, 238, 239]
PDAPP	Swiss Webster × B6D2F1	Human APP (Indiana)	Aβ plaques at 6-9 months	Synaptic loss.	Behavioral impairment in novel object recognition at 6 months, Morris water maze at 3 months.	[54, 240]
J20	C57BL/6 × DBA/2 F2	Human APP (Swedish and Indiana)	Diffuse amyloid deposits at 5-6 months and larger neuritic plaques at 9 months	Phospho-neurofilaments.	Behavioral impairment in novel object recognition at 4 months, Morris water maze at 6-9 months.	[53, 68, 241, 242]
TgCRND8	C3H/He × C57BL/6	Human APP695 (Swedish and Indiana)	Aβ plaques at 3 months,dense core plaques at 5 months, spreading to the cerebellum and brainstem by 8-9 months	Astrocytic gliosis and microglial activation in regions around plaques.	Behavioral impairment in novel object recognition at 3-5 months, Morris water maze at 3 months.	[67, 69, 243]
*App*^NL-F^	C57BL/6	Human APP (Swedish and Iberian)	Aβ plaque at 6 months	Synaptic loss, microgliosis and astrocytosis	Behavioral impairment in Y-maze at 18 months, no impairment in Morris water maze at 18 months	[76]
5XFAD	Tg6799×Tg7031 ×Tg7092	Human APP (Swedish, Florida, London); Human PS1 (M146L, L286V)	Intraneuronal Aβ42 accumulation at 1.5 months, amyloid deposition, gliosis, at 2 months	Significant neurodegeneration and neuronal loss.	Behavioral impairment in Y-maze at 4-5 months, decreased Interest in social-related behaviors at 3-12 months. Morris water maze at 4 months.	[81, 244, 245]
APP23 x PS1-R278I	C57BL/6J	Human APP23 (Swedish K651N, M652L); Human PS1 (R278I)	Aβ plaque at 6 months	Astrocytosis.	Behavioral impairment in Y-maze at 3-4 months; no significant impairment in Morris water maze	[82]
TREM2-BAC x 5XFAD	TREM2-BAC: FVB/NJ; 5XFAD: C57BL/6X SJL	Human APP (Swedish, Florida, London); Human PS1 (M146L, L286V)	less cortical amyloid plaque at 7 months compared to 5XFAD mice	Enhanced process ramification and phagocytic marker expression in plaque-associated microglia; improved dystrophic neurites.	No cognitive impairment in contextual fear conditioning test.	[85]
3xTg-AD	C57BL6/129SvJ	Human APP (Swedish); Human PS1 (M146V); Human Tau (P301L)	Aβ plaque at 6 months	synaptic dysfunction and increased microglia activation at 6 months; Tau alteration at 12-15 months	Retention deficits in Morris water maze and contextual fear memory	[87-89]

**Figure 2.**
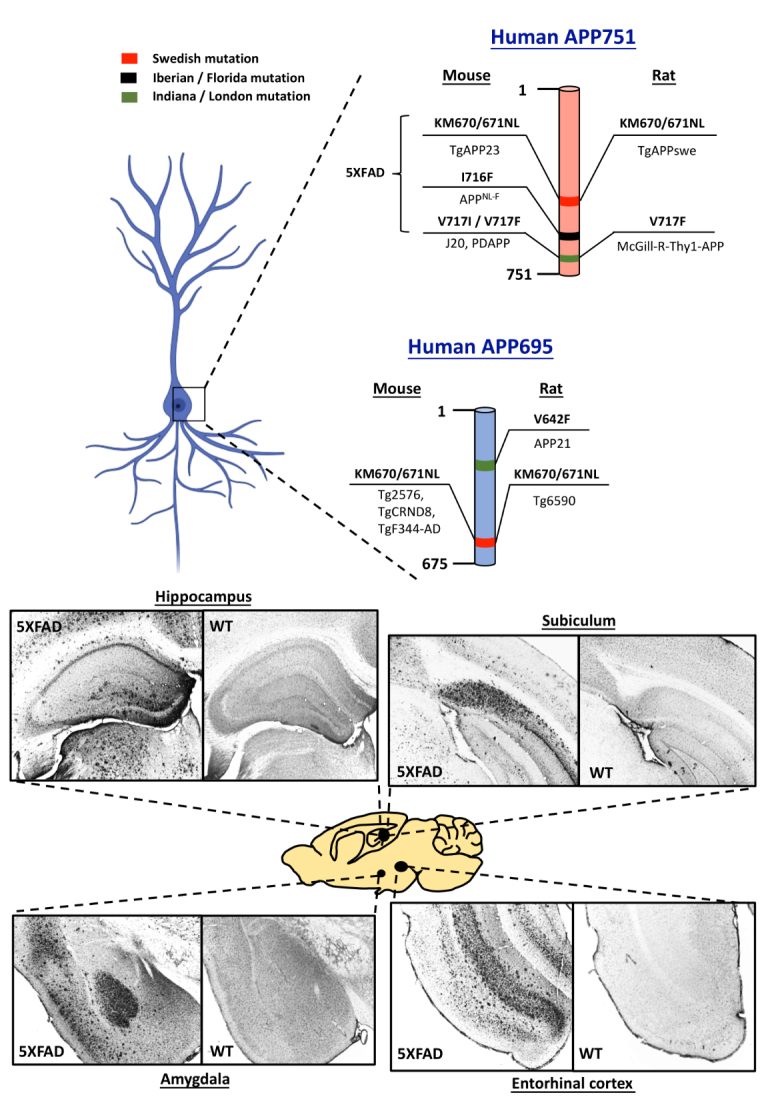
Human APP695 and APP751 are essential genes involved in the generation of transgenic rodent models of Alzheimer’s disease (AD). Swedish (red), Iberian / Florida (Black) and Indiana / London (Green) are the major mutations introduced into the human APP in rodents to induce rapid amyloid plaque formation. One of the rodent models that encompass numerous mutations is 5XFAD, 3 mutations of which are located on human APP751. Representative photomicrographs showing intense amyloid plaque formation in various brain regions demonstrate severe Aβ pathology in 5XFAD mouse model in the age of 6 months. These images highlight the Aβ pathological progression in brain regions established for memory processes.

The findings from studies using transgenic rats suggest they could be used in the structural analysis of the AD brain. Moreover, given their larger size compared to mice, rats would be a better choice for complicated procedures, such as the intraventricular injections, acquisition of cerebrospinal fluid, electrophysiology, neuroimaging, and neurosurgical measures [120]. In addition, their size also allows fine molecular manipulation to be performed *in vivo*. In terms of their behavior, rats exhibit more complex social behaviors, including juvenile fighting, courtship behaviors, and low-level aggressive behaviors [121]. Notably, rats have been shown to be less stressed in some behavioral memory paradigms that utilize water such as the MWM [121]. A stable mental state is essential for valid and reliable behavioral outcomes in behavioral testing of animals.

Despite the lack of animal models that exhibit disease development fully resembling that of human AD, the abovementioned animal models were found to consistently reflect the pathology of Aβ accumulation and/or amyloid plaques formation. The question of whether Aβ is the sole driving force in AD pathogenesis remains unanswered. Thus, further investigations using a well-establish animal model that encompasses the major AD pathologies is still needed. Nonetheless, with advancements in current genetic research and disease models, we are optimistic that a more comprehensive model can be developed that can elucidate the underlying relationship between Aβ and AD.

## Limitations of rodent models in AD

Although research using animal models has yielded promising results, their clinical translational value remains ambiguous. Currently, 132 agents have been evaluated in clinical trials, including 88 agents in 42 phase 3 trials, 74 agents in 83 phase 2 trials, and 30 agents in 31 phase 1 trials [122]. In disease modification trials of 96 agents, 38 agents focused on amyloid as the primary target or one of the main targets [122]. In particular, amyloid was the most common target of disease modification studies in phase 2 and 3 trials [122]. Although many agents have been evaluated in clinical trials, none of them has yet to be applied as a clinical treatment. The possible underlying reasons for the failure of these agents include (i) a lack of understanding of the neurobiological basis of AD resulting in the neuroprotective treatment failing to target crucial factors, (ii) the inability of current AD animal models to completely mimic AD pathology resulting in incompatible findings between preclinical and clinical phases, and (iii) insufficient treatment length in the trials, considering AD is a chronic neurodegenerative disease that may require long-term pharmacological treatment and regular monitoring. Furthermore, strict regulations should be implemented for testing drugs to maintain high safety and low side effects, and the risk/benefits should also be fully considered. However, all these factors cannot be completely addressed by preclinical animal models, and there are certain limitations in the use of transgenic animal models in AD research.

The effectiveness of a transgenic animal model in mimicking certain diseases relies heavily on how they respond in behavioral assessments. Behavioral findings from many studies employing transgenic rat models are relatively reliable, as the behavioral tests demonstrate rats have consistent behavior. However, we observed varying results from studies using AD mouse models. This phenomenon may stem from the emotional vulnerability of mice to stress and novel environments, which hinders their normal cognitive ability during testing. Furthermore, some rodent models did not show cognitive declines in the behavioral testing, which questions the validity of these models. This warrants further investigation with a more controlled behavioral testing paradigm. Most of the studies employed rodent models that exhibited increased Aβ levels and/or plaque formation as the only disease manifestation, which does not reflect the full pathology of AD. For example, Tg2576 and PS1/APP mouse models failed to mimic the neuron loss, which is one of the hallmarks of AD [55, 123]. Moreover, the age and gene background of the animal model as well as the experimental conditions can greatly impact the research outcomes [124]. This highlights the importance of more reliable neuroscientific methodology in disease research.

Another hypothesis of Aβ plaque formation suggests that Aβ accumulation is a defense mechanism against other pathologies including cellular dysfunction and dysregulation, but this has yet to be fully investigated [124]. Given the contradictory role of Aβ, researchers should consider the advantages and limitations of the different animal models and choose the most appropriate one.

## Exploring therapeutic options using rodent models of AD

Currently, treatments only focus on alleviating major disease symptoms of AD. However, these treatments become ineffective in the later stages of AD as the severity of the disease has escalated. Due to the late manifestation of symptoms and ethical constraints, it is difficult to conduct accurate clinical evaluations in AD patients. Thus, animal models can provide valuable information to elucidate the pathological progression of AD and facilitate the discovery of potential pharmacological treatments that may later translate to the clinical setting. In this section, we discuss components of AD pathology as possible viable targets for disease modulation.

### Neuroinflammation

It was generally accepted that the brain was immunologically privileged due to the presence of the blood-brain barrier (BBB), which prevents immune cells and humoral factors from entering the nervous system. It was also generally recognized that brain cells were incapable of eliciting an immune response. However, this idea was challenged by several discoveries in the past two decades, in that disruption of the BBB was consistently reported in AD cases [125]. Epidemiological investigations have revealed the role of neuroinflammation in the brain. Likewise, research has shown that brain tissues can change their innate paracrine systems through autonomously-generated and regulated inflammatory molecules [126]. Given the role of inflammation in the pathogenesis of brain diseases such as AD, several anti-inflammatory treatments have been investigated. For example, chronic administration of non-steroidal anti-inflammatory drugs (NSAIDs) was found to reduce the risk of developing AD by 80% in populations over 55 years of age without a history of dementia prior to evaluation [127]. Although neuroinflammation is an integral factor in AD pathogenesis, it is important to identify the agents that play a major role in this process.

Actrocytes are an important component in the process of neuroinflammation. Reactive astrogliosis, which is a multi-phase remodeling of astrocytes, is beneficial for neuronal protection and recovery of impaired neural tissues [128, 129]. In addition to activated microglia, activated astrocytes, which become hypertrophic, gradually accumulate and surround senile plaques in the brains of AD patients. Activation of glial cells can also occur in the early stage of AD, sometimes even prior to the formation of Aβ deposition [130]. Although reactive astrocytes are characterized by their functional decline and increased expression of glial fibrillary acidic proteins [131], their domain organization was found to be maintained and there was no evidence of scar generation. In animal models of AD, early features include astroglial atrophy, which may significantly reduce synapse coverage and possibly underlies the impaired neurotransmission and cognitive deficits observed in AD [33, 131-134]. Furthermore, this atrophy suggested a spatio-temporal pattern of disease progression, with signs of atrophy first observed in the area of entorhinal cortex in early AD and subsequently affecting astrocytes located far away from the senile plaques in late AD. Similar to microglia, when astrocytes are exposed to Aβ, they start to release cytotoxic molecules such as cytokines, interleukins, and NO. Such reactions then aggravate the neuroinflammation throughout the course of AD. Previous studies in an animal model of AD, which employed adeno-associated virus-driven suppression of the molecular cascade that indirectly inhibited astrocyte activation, revealed they had decreased Aβ deposition [135]. Such suppression also restored cognitive abilities and reduced astrogliosis [135], indicating that astroglial activation could be modulated to alleviate this disease. Moreover, the clearance of Aβ induced by astrocytes was dependent on apoE60, and astrocyte-associated lipidation might enhance other mechanisms of Aβ clearance such as microglia-mediated elimination of Aβ [136, 137]. Indeed, astrocytes were found to promote internalization and degeneration of Aβ in *in vitro*experiments [138]. The above evidence suggests that astrocytes are essential for the clearance of Aβ.

Microglia are widely distributed in the central nervous system (CNS) and are known to scavenge and eliminate cellular debris or external pathological agents in specific brain areas. They can also support tissue maintenance and plasticity of neural circuits by supplying neurotrophic factors [139], and protect and remodel synapses [140]. Upon activation, microglia spread and start to migrate to the specific injury location, which triggers the innate immune response. Microglia bind to soluble Aβ oligomers and Aβ fibrils through receptors, including class A scavenger receptor, CD36, and toll like receptors (TLR types 4 and 6) [141-144], which activates microglia to begin forming proinflammatory cytokines and chemokines [143, 145] to initiate an inflammatory response. The secretions of proinflammatory cytokines and chemokines are CD36- and TLR-dependent, as deletion of these two genes *in vitro* decreased the generation of Aβ-induced cytokines [143, 145, 146] and prevented the accumulation of intracellular amyloid and activation of inflammasomes [146]. Despite initiating neuroinflammation, microglia in fact also exhibit great efficacy in suppressing amyloid deposits [147]. Specifically, blood-derived microglia were able to eliminate amyloid deposition by phagocytosis via direct binding of microglia to Aβ [147]. Another study showed that the accumulation of microglia prior to Aβ elimination was mediated by its chemokine receptor *Ccr2*,the depletion of which induced increased Aβ deposition [148]. Additionally, Tg2576, a transgenic animal model of AD, displayed high premature fatality upon *Ccr2* deficiency, which further implies that *Ccr2*-dependent microglial Aβ clearance may exert neuroprotective effects in early AD. These findings further demonstrate the bidirectional consequences of microglial activation in the brain, given its roles in Aβ clearance and neuro-inflammation.

Given that neuroinflammation has a significant role in AD, much effort has been devoted to researching the therapeutic effects of anti-neuroinflammation in AD. Indeed, several epidemiological studies indicate that long-term administration of NSAIDs can reduce the risk of developing AD [149, 150]. Preclinical studies on the use of NSAIDs in AD models have provided promising results, including reduction of Aβ secretion and deposition, γ-secretase activity regulation, anti-inflammatory effects, and enhance cognitive functions in rodent models [151-156]. However, the translational value to the clinic remains ambiguous, as clinical evidence substantiating the therapeutic effect of NSAIDs is still lacking [157].

Besides NSAIDs, other strategies focusing on anti-neuroinflammation have been studied, including peroxisome proliferator-activated receptor (PPAR)-γ agonists and antibodies that inhibit TNFα signaling. Interestingly, administration of a commonly prescribed diabetes drug, pioglitazone, which is also a PPAR-γ agonist, was associated with reduced dementia risk in patients who had non-insulin-dependent diabetes mellitus [158]. The PPAR-γ agonists were found to decrease the generation of inflammatory cytokines and amyloid deposit in AD rodent models [155, 159]. Specifically, rosiglitazone was able to rescue cognitive deficits observed in the Tg2576 mouse model, possibly through ERK pathway activation [160, 161]. Increased TNFα, which is highly implicated in neuroinflammation and cell death processes, was consistently observed in both preclinical and clinical cases. Deletion of its receptor contributed to diverse effects including BACE1 activity suppression and restoration of cognitive abilities [162, 163], suggesting TNFα signaling could have a modulatory role in alleviating AD. Indeed, inhibition of TNFα signaling by antibodies against TNFα, such as infliximab, significantly reduced Aβ plaque formation and tau phosphorylation in APP/PS1 mice [164], further supporting TNFα inhibition as a viable therapeutic approach.

Although neuroinflammation is considered to be involved in AD pathogenesis, there are many unanswered questions: What is the exact mechanism of neuroinflammation in AD? Is neuroinflammation a factor that triggers or aggravates the course of AD? To what extent does it affect the onset or progression of AD? Further studies are needed to delineate the underlying mechanisms and crucial cellular players of neuroinflammation in AD.

### Neurotrophic factors

Neurotrophic factors play crucial roles in neuronal nourishment, survival, and regeneration. Previous evidence suggest they are also involved non-neuronal tissue survival, proliferation, differentiation, and anti-inflammation processes. Additionally, they have been detected in tissue-specific adult stem cell niches involved in tissue regeneration outside of the nervous system [165-167]. There has been some research into using neurotrophic factors as a treatment for AD. Neurotrophic factors were subcutaneously injected to generate a systemic exposure in an amyotrophic lateral sclerosis clinical trial [168, 169]. However, such an administration route was demonstrated to induce significant side effects. Indeed, one of the challenges encountered was the need to enhance the specificity of the delivery of neurotrophic factors to the targeted neurons in the brain. Researchers employed intrathecal administration of neurotrophic factors, such as brain-derived neurotrophic factor (BDNF) and ciliary neurotrophic factor (CNTF), to focus the delivery to the CNS. However, analysis of lumbar and cervical taps showed the injected neurotrophic factors failed to reach the spinal cord and brain. Dose-dependent side effects were also reported after intrathecal administration of neurotrophic factors [170, 171]. These results indicate further improvements in the delivery of these therapeutics is still needed.

Gene therapy is a novel technology that can enable controllable and predictable expression of long-term biologically active proteins with high spatial specificity [172]. Previous clinical trials used this technology to genetically modify autologous fibroblasts to express human nerve growth factor (NGF), which were delivered to the basal forebrain of AD patients. The treatment was able to improve cognition and metabolic activity in cortical areas of AD patients, which supports the use of neurotrophic factor as an effective therapeutic agent for AD [173]. Similarly, astrocytic glial cell-line derived neurotrophic factor (GDNF) overexpression in the hippocampus induced by recombinant lentiviral vectors was able to restore cognitive function in a 3xTg-AD mouse model and aged rats [174, 175]. These studies demonstrate the crucial role of neurotrophic factors in the alleviation of AD symptoms. In particular, BDNF has a prominent role in facilitating neuronal survival and differentiation, neurotransmitter release, dendritic remodeling, and axonal growth [176]. Given that BDNF is essential in mediating synaptic plasticity that serves as the cellular basis of learning and memory functions in adults, BDNF may be involved in the progression of the cognitive decline in AD [177]. Acetylcholinesterase (AChE) inhibitors are recognized as a preferred treatment option for AD, and drugs such as donepezil and galantamine have been used in early AD. Previous studies showed that restoration of BDNF levels could be induced by these two drugs in AD patients and animal models [178-180]. This may suggest that the neuroprotective effects exerted by AChE inhibitors in AD may be mediated by the BDNF signaling pathway. However, chronic galantamine treatment (3 mg/kg, i.p., 14 days) failed to alter hippocampal BDNF levels in mice, but this result may not applicable because of differences in the physiology between wildtype and AD mice. Other treatments including antidepressant, phosphodiesterase inhibitors and small-molecule BDNF mimetics can enhance BDNF levels in AD. Previous clinical studies reported Cerebrolysin, a mixture of neurotrophic factors extracted from pig, had therapeutic effects as demonstrated by a persistent enhancement after short-term treatment in AD [181, 182]. In addition, Cerebrolysin-treated apoE-ko mice had improved behavioral impairments and alleviated neurodegeneration [183]. To date, much effort has been made to explore ways to restore BNDF levels in the brain of AD patients, but there are several challenges that need to be overcome including drug biostability, BBB permeability, optimal dose, administration time, and specificity of drug molecules in diseased neurons. Further studies are needed to optimize the treatment paradigms.

### Neuroplasticity and neurogenesis

Neuroplasticity is regarded as the fundamental mechanism in learning and memory and also the brain’s ability to respond to neuronal injury. Neuroplasticity is a constant process in response to neuronal activity, injury, death, and neurogenesis, as well as the regulation of structural and functional processes related to axons, synapses, and dendrites. Plasticity involves various structural processes, such as synaptic function, synaptic remodeling, synaptogenesis, neurite extension, and neurogenesis. Although the response of the central nervous system has been shown to be mediated by neuroplasticity, its effects can be limited. Given that impaired neuroplasticity is observed in AD, stimulating neuroplasticity may show promise in alleviating symptoms in AD [184].

Adult hippocampal neurons possess the ability to proliferate [185, 186] and persistently replace neurons in the area of the dentate gyrus [186], especially during learning activities [187], which suggests that adult neurogenesis is essential for cognitive behavior. Neurogenesis has been shown to decline in hippocampal areas with age [188]. Restoring neurogenesis by replenishing neural stem cells and replacing lost neurons could be an effective approach in the development of AD treatments. Such methods may be appropriate as neurogenesis naturally occurs, and thus replacement of neurons will not be affected by the stimulation of abnormal sprouting, although evidence supporting the substitution of functions in new neurons is insufficient. The most important issue is to maintain the functions of the transplanted cells, including orchestration of topographically precise migration, directed differentiation, and synapse functionality [184, 189, 190]. Peripheral injection of multipotent cells was shown to migrate from the blood lineage, which may be an approach to replacing lost neurons in the CNS [191]. While this theory may seem promising, more studies on this treatment paradigm using appropriate animal models need to be conducted to validate its effectiveness.

Neurotrophic factors serve as a prominent mediator of neurogenesis and neuroplasticity. In particular, NGF can stimulate sprouting and outgrowth when administered after onset of the cognitive impairment [192]. The responsiveness to NGF partly depends on tropomyosin receptor kinase A (TrkA) receptor expression, and was demonstrated to underlie synaptic plasticity via modulation of acetylcholine release [193, 194]. This finding indicates that NGF dynamics may be crucial to neurological functions, given the importance of cholinergic neurons in the CNS. Indeed, NGF was shown to be a crucial regulator of neuronal morphology and function, which can maintain or promote cholinergic function in AD through improving the survival of degenerating neurons, improving sprouting, promoting neurotransmitter synthesis, and promoting neuron firing [195]. Application of NGF in unimpaired CNS leads to enhanced growth and sprouting of cholinergic neurons, and increased choline acetyltransferase level and choline uptake [196-198]. In adults, the need for NGF in cholinergic neuron survival is still debatable [195]. Surprisingly, increased NGF was found in the cortex and hippocampus of AD brains [199-201], which may indicate the declined capacity of NGF retrograde transport in cholinergic neurons does not meet the increased demands of cholinergic input to possibly compensate the deficits in AD.

Recently NGF treatment for 12 months in AD patients resulted in cognitive improvements and reduced Aβ levels in the cerebrospinal fluid [202]. However, patients responded to such treatment paradigm exhibited signs of brain atrophy, which was indicated by enhanced phosphorylated tau [202]. Moreover, some subjects reported side effects such as back pain, suggesting lower doses of NGF or other alternative routes may be needed to optimize the treatment [203].

### Aβ inhibition / clearance

Currently, research on neuroprotective treatments for AD have focused on the amyloid hypothesis, in which abnormal metabolism of APP leads to the formation of toxic Aβ species and subsequent manifestation of the pathological features of AD. Based on this hypothesis, one treatment strategy would be to administer compounds that can block the generation or increase the clearance of Aβ42 to alleviate disease progression. Currently, 32% of agents under phase 3 trials are anti-amyloid treatments, including immunotherapy (n=6), BACE inhibition (n=2), and anti-aggregation (n=1). There are currently four monoclonal antibodies against Aβ in phase 3 trials: Aducanumab [204], Crenezumab [205], Gantenerumab [206], and Solanezumab [122, 207]. Such antibodies bind to specific types of Aβ aggregates: Aducanumab and Gantenerumab tend to bind to Aβ aggregates; Solanezumab binds to soluble Aβ monomers; and Crenezumab and Bapineuzumab have high affinity for Aβ oligomers [208]. Several short peptides and peptidomimetics have also been used to target Aβ to prevent aggregation [209]. However, the Aβ binding sites of these agents are still unclear, as they differ from monoclonal antibody binding regions mentioned above [210-212].

As novel AD treatments, anti-Aβ immunotherapy has the potential to reduce the pathological injury and improve cognitive decline, but they still need clinical trials. Active AD immunization or vaccination can be achieved by introducing Aβ peptide fragments and adjuvant to trigger an immune response, leading to the generation of antibodies against Aβ and removal of the Aβ plaques [213]. In a randomized placebo-controlled study, mild to moderate AD patients were administered anti-Aβ immunotherapy for 48 weeks. After the treatment period, the intervention group did not have significantly higher disability assessment scores compared to the control group. However, after 2 years, the phase 2 trial showed the intervention group had significant amyloid plaque clearance, although some patients developed aseptic meningoencephalitis and were terminated from the trial [214]. A possible reason for this adverse reaction might be due to adjuvant-mediated T helper 1 cells infiltration into the CNS, leading to autoimmune neuritis. Other side effects of Aβ immunotherapy can include amyloid-related imaging abnormalities (ARIA) [215]. Such side effects should be taken into account when assessing the trials.

### Pathways other than Aβ

During the process of developing a new drug, a crucial step is to validate its therapeutic effect in animal models of the disease. The selected animal models should adequately mimic the crucial pathology of the disease. If the therapeutic effect is achieved in these animal models, then the drug might proceed to the next step of clinical trials. Current mouse models of AD have single or double mutations of FAD APP and/or PS1. Although these mouse models may display Aβ plaques, other significant hallmarks of AD such as neurofibrillary tangles and neuronal loss are not present [216]. Therefore, drugs tested in such animal models may fail to achieve validity, complicating the translation of preclinical results to the clinical setting. Given that animal models based on the amyloid hypothesis do not fully reflect the pathophysiology of AD, other pathologies might need to be considered. Here, we briefly highlight current advances in therapeutic agents that target other pathologies besides Aβ plaques.

Another well known hypothesis of AD is the Tau hypothesis, in which hyperphosphorylation of tau protein is a crucial event in AD pathology. Tau is a cytoplasmic protein that binds to tubulin and stabilizes microtubules to maintain cytoskeleton integrity under normal cellular physiology. Hyperphosphorylated tau leads to the formation of neurofibrillary tangles and neuropil threads that disrupt this integrity, making tau a possible target for new treatments [217]. Various agents have been designed to remove tau, prevent tau propagation, and reduce tau phosphorylation and/or neurofibrillary tangle formation. There are eight such agents currently undergoing phase 2 clinical trials, including AADvac1 [218], ABBV-8E12 [219], BIIB092 [220], LY3303560 [221], and Methylene blue [122, 222]. At present, only one compound TRx0237(LMTX) that may reduce tau-mediated neuronal damage (DMT) is undergoing phase 3 clinical trials, which is still currently recruiting subjects [223].

Besides the above two hypotheses, recent evidence indicates a possible link between mitochondrial dysfunction and AD [224]. It is perhaps not surprising that drugs that can restore impaired mitochondrial functions in neurodegenerative disorders such as Parkinson’s disease are now undergoing rapid development as treatments for AD. Drugs, such as Latrepirdine (Dimebon), which was shown to have neuroprotective properties in rescuing Aβ-induced mitochondrial impairment, have been developed for clinical use [225]. However, a phase 3 clinical trial found this drug did not improve the two endpoints (Alzheimer’s disease Assessment Scale-cognitive Subscale and Alzheimer’s disease Co-operative study-Activities of Daily Living Inventory) when compared to the placebo group [226].

Interventions based on RNA (antisense oligonucleotide or interference RNA), such as therapies for transthyretin-mediated amyloidosis, may have potential in treating neurological diseases. Oligonucleotide-based treatments that have shown initial effects in Huntington’s disease could be applied to other neurodegenerative disease such as AD [227].

Other treatments that have been in phase 3 trials include an omega-3 polyunsaturated fatty acid (docosahexanoic acid; [228]) and vitamin E (α-tocopherol; [229]), both with and without the NMDA receptor antagonist memantine. The former was shown to have no clinical effects [230], but the latter antioxidant vitamin E is still controversial. The debate is still ongoing about the form of vitamin E and its combination treatments. Its efficacy and safety were assessed in the Selenium and vitamin E Cancer prevention trial (SELECT) for prostate cancer [231]. Cholesterol-lowering medications, such as HMG-CoA reductase inhibitors (statins) that are used to treat vascular disease, have also be considered as a treatment for AD or to reduce the risk of developing AD. However, two statins, atorvastatin [232] and simvastatin [233], showed no clinical benefit in phase 3 trials in AD patients [234]. A multitargeted molecule, GV-971, which showed preclinical improvements on amyloid plaques, neurofibrillary tangles, mitochondrial function, neuroinflammation and cholinergic function, has completed a phase 3 trial in China in 2018 [235], and a phase 1 investigation of its pharmacokinetics and safety doses is conducted as well [236]. The agent is currently under review by Chinese FDA. Overall, given the promising findings in preclinical studies, further clinical studies are warranted to substantiate its seemingly beneficial effects.

## Conclusion

Numerous transgenic rodent models have been generated in the past two decades in the attempt to further the research on AD pathogenesis. Transgenic rodent models that display Aβ plaque formation are valuable tools to address unanswered question about Aβ pathology in AD. Development and optimization of such models will provide an ideal platform for studying disease progression and for screening therapeutic options. Although these rodent models fail to mimic all pathologies of human AD, they are indispensable in medical research as they allow terminal experiments/procedures to investigate underlying molecular mechanisms. Thus, they continue to play an important role in advancing our understanding of Aβ pathology and in evaluating the efficacy and safety of potential medicines for AD.
